# Prognostic value and outcome for acute lymphocytic leukemia in children with MLL rearrangement: a case-control study

**DOI:** 10.1186/s12885-022-10378-w

**Published:** 2022-12-02

**Authors:** Kun-yin Qiu, Dun-hua Zhou, Xiong-yu Liao, Ke Huang, Yang Li, Hong-gui Xu, Wen-jun Weng, Lu-hong Xu, Jian-pei Fang

**Affiliations:** 1 Children’s Medical Center, Sun Yat-sen Memorial Hospital, Sun Yat-sen University, Guangzhou, 510120 People’s Republic of China; 2 Guangdong Provincial Key Laboratory of Malignant Tumor Epigenetics and Gene Regulation, Sun Yat-Sen Memorial Hospital, Sun Yat-Sen University, Guangzhou, 510120 People’s Republic of China

**Keywords:** MLL, Children, Acute lymphoblastic leukemia, Outcome, Prognosis

## Abstract

**Purpose:**

To evaluate the prognostic factors and outcome for acute lymphoblastic leukemia (ALL) in children with MLL rearrangement (MLL-r).

**Methods:**

A total of 124 pediatric patients who were diagnosed with ALL were classified into two groups based on the MLL-r status by using a retrospective case-control study method from June 2008 to June 2020.

**Results:**

The prevalence of MLL-r positive in the whole cohort was 4.9%. The complete remission (CR) rate on Day 33 in the MLL-r positive group was not statistically different from the negative group (96.8% vs 97.8%, *P* = 0.736). Multivariate analysis showed that T-cell, white blood cell counts (WBC) ≥ 50 × 10^9^/L, MLL-AF4, and D15 minimal residual disease (MRD) positive were independent risk factors affecting the prognosis of MLL-r positive children. Stem cell transplantation (SCT) was a favorable independent prognostic factor affecting event-free survival (EFS) in MLL-r positive patients (*P* = 0.027), and there was a trend toward an independent prognostic effect on overall survival (OS) (*P* = 0.065). The 10-year predicted EFS for patients with MLL-AF4, MLL-PTD, MLL-ENL, other MLL partner genes, and MLL-r negative cases were 46.67 ± 28.61%, 85.71 ± 22.37%, 75 ± 32.41%, 75 ± 32.41%, and 77.33 ± 10.81%, respectively (*P* = 0.048). The 10-year predicted OS were 46.67 ± 28.61%, 85.71 ± 22.37%, 75 ± 32.41%, 75 ± 32.41%, and 85.2 ± 9.77%, respectively (*P* = 0.049). The 124 patients with ALL were followed up and eventually 5 (4%) cases relapsed, with a median relapse time of 3.9 years.

**Conclusion:**

Patients with MLL-r positive ALL have moderate remission rates, but are prone to relapse with low overall survival. The outcome of MLL-r positive ALL was closely related to the partner genes, and clinical attention should be paid to screening for MLL partner genes and combining them with other prognostic factors for accurate risk stratification.

**Supplementary Information:**

The online version contains supplementary material available at 10.1186/s12885-022-10378-w.

## Introduction

The mixed lineage leukemia (MLL) gene, located on the long arm of chromosome 11, region 2, band 3 (11q23), which is fused to a variety of translocation partner genes (TPG), is known to identify 135 different MLL rearrangements, and its rearrangements produce MLL fusion proteins can cause abnormal self-renewal and epigenetic deregulation of hematopoietic progenitor cells, leading to the development of malignant leukemia [1]. Previous literature showed that the prevalence of MLL gene rearrangement (MLL-r) in childhood acute lymphoblasitc leukemia (ALL) is 2.5 to 5% [2, 3]. Compared with non-MLL leukemia, children with MLL-r positive ALL have clinical features that are different from other types of leukemia, mainly high white blood cell count (WBC) at disease onset, insensitivity to traditional chemotherapeutic agents, low complete remission rate (CR), and short survival and poor outcome [4–6], which is why the South China Children’s Leukemia Group [7] classified children with MLL-r positive ALL as a high-risk group.

The children with ALL who have fused the N-terminal gene and any of the different partner genes to produce a new MLL fusion protein within the framework combine with different fusion partner genes [5]. The prognosis is different, but the prognosis is usually poor. MLL fusion gene is related to age and sex. The incidence rate of MLL-AF4 in infants and adults is higher than that in children, the incidence rate of MLL-ENL in infants and children is higher than that in adults, the incidence of AF10 in children is higher, and PTD tends to children and adults. MLL-AF10 appears more frequently in male patients, while female patients are more affected by MLL-AF4 fusion [4–7].

Currently, although a number of studies have reported MLL-r positive ALL, there are only a few literature reports focusing on partner genes. Hence, in this study, we collected data from 637 children with ALL in our hospital over the past ten years, tested MLL gene rearrangements and their partner gene types, retrospectively analyzed the clinical characteristics and laboratory data of children with MLL-r positive ALL, and evaluate the outcome and prognostic value of this type of children.

## Patients and methods

### Study participants

All cases were enrolled from 637 children with ALL who were initially diagnosed in the Department of Hematology at our Children’s Medical Center from June 2008 to June 2020, and 31 cases were included in the MLL-r positive group (case group) and 93 cases from four common fusion genes (ETV6/RUNX1, BCR-ABL1, MLL gene rearrangement, and E2A-PBX1 fusion gene) were negative. The 93 children were randomly selected by systematic sampling from 465 children with ALL as the MLL-r negative group (control group). The systematic sampling method was as follows: 465 children with ALL were numbered 1–465 according to the time of diagnosis, in groups of 5, and a random number 2 was generated by applying a random number list, and 93 cases were selected from 2, 7, 12, 17, 22, 27, 32, 37, 42, 47, 52, and so on to 465 to form the control group. The study was conducted in accordance with the principles set down in the Declaration of Helsinki and was approved by the Ethics Committee of Sun Yat-sen Memorial Hospital. All the patients’ parents or guardians, provided written informed consent.

Clinical characteristics such as age, gender, WBC, Hemoglobin (Hb), platelet (PLT), risk stratification and immunophenotype at the time of initial diagnosis were analyzed in the whole cohort, and the response to prednisone treatment, BM smear and minimal residual disease (MRD) on Day 15 and Day 33 of induction chemotherapy were also monitored.

Inclusion criteria: ① Age ≤ 18 years; ② Clinical presentation consistent with ALL and definite diagnosis of ALL by BM cytomorphology, immunophenotype, cytogenetics and molecular biology (ETV6/RUNX1, BCR-ABL1, MLL gene rearrangement and E2A-PBX1 fusion gene as routine tests); ③ First-episode children (not undergoing any ALL before coming to the hospital for related treatment). All the patients were screened for relevant fusion genes by FISH (fluorescence in situ hybridization) or quantitative PCR (Polymerase Chain Reaction) at the time of initial diagnosis.

Exclusion criteria: (i) T-lineage, mature B or acute mixed leukemia; (ii) Secondary to immunodeficiency disease; (iii) Second tumor; (iv) Definite CML (chronic myelocytic leukemia) acute change; (v) ALL with Down’s syndrome; (vi) Glucocorticoid use for more than 1 week in the month before enrollment; (vii) MLL data missing.

### Chemotherapy protocol

Children diagnosed between February 2008 to September 2016 were treated according to the Guangdong Children’s Leukemia Group-ALL-2008 (GD-ALL-2008) [8] protocol; and children diagnosed between October 2016 to June 2020 were treated according to the South China Children’s Leukemia Group-ALL-2016 (SCCLG-ALL-2016) [7] protocol.

### Treatment response

Prednisone treatment is beneficial to risk stratification and prognosis evaluation of ALL patients. ① Prednisone response (PR): those with absolute peripheral blood naive lymphocyte count < 1000 × 10^6^/L on Day 8 of prednisone induction were considered prednisone good responders (PGR), while those ≥1000 × 10^6^/L were considered prednisone poor responders (PPR); ② Morphological evaluation of bone marrow smear was performed on Day-15 and Day-33. Patients were classified according to their blast cells amount, with M1 (blast cells< 5%), M2 (5% to< 25%), or M3 (> 25%); (3) Relapse: including BM relapse, central nervous system (CNS) relapse, testicular relapse and combined relapse.BM relapse means that after complete remission (CR) of ALL, the ratio of primitive plus naive cells in bone marrow is > 25% on review. CNS relapse means that primitive plus naive cells are detected in cerebrospinal fluid by centrifugal smear or CNS infiltration is present and other causes cannot be explained or CT/MRI showed brain or meningeal lesions, testicular relapse i.e. ultrasound or biopsy confirmed unilateral or bilateral testicular leukemic cell infiltration, combined relapse i.e. extramedullary relapse (central and/or testicular relapse) with > 5% leukemic cells in the BM; ④ Events included: persistent non-CR, relapse, development of a second tumor, death and so on.

### Risk classification

Stand-risk (SR) group: Patients with B-cell precursor ALL were (1) between 1and 6 years of age and had a WBC count at diagnosis < 20 × 10^9^/L and (2) show a good early response, including a good response to prednisone on day 8 or M1/M2 marrow on day 15 and (3) M1 marrow on day 33.

Intermediate-risk (IR) group: Patients had (1) T-lineage ALL or below 1or after 6 years of age and had a WBC count at diagnosis>20 × 10^9^/L or (2) show a good early response, including a good response to prednisone on day 8 or M1/M2 marrow on day 15 and (3) M1 marrow on day 33 (4) CNSL (central nervous system leukemia).

High-risk (HR) group: Patients who had (1) show a poor early response, including a poor response to prednisone on day 8 or M3 marrow on day 15 or (2) M2/M3 marrow on day 33 or (3) t(9:22)(BCR/ABL) or had t(4:11) MLL/AF4 or (4) Testicular leukemia. The final risk group was determined based on the treatment response.

### Follow-up

All cases were followed up by outpatient review or telephone, and children receiving the GD-ALL 2008 protocol were followed up until June 30, 2018, while children receiving the SCCLG-ALL 2016 protocol were followed up until July 31, 2020, with study endpoints set as death, lost to follow-up, or follow-up cutoff, and those lost to follow-up. The overall survival (OS) period was the time from the start of treatment to death or the end of follow-up. The event-free survival (EFS) period was the time from the start of treatment to the occurrence of any event, including death from any cause, second tumor, disease progression, relapse, or missed follow-up. Patients were observed for general condition, relapse and interventions, survival, and monitoring of BM.

### Statistical analyses

Baseline characteristics were grouped by MLL status and presented as mean ± SD for continuous variables and as frequency (%) for categorical variables. Chi-square test was used for categorical variables, when the number of samples is small, we use Fisher’s exact test. In addition, analysis of variance or Kruskal-Wallis test was used for continuous variables. In order to assess the clinical outcome, we used the following concepts: complete remission (CR, was defifined as less than 5% lymphoblasts in active hematopoietic bone marrow at the end of induction), event-free survival (EFS, defined as the start of the study to the timing of events for the first time, including induced failure, progress, or any cause of death and recurrence), and overall survival (OS, defined as the time between the beginning of learning and death from any cause). Cox proportional hazards models were used to test the associations between EFS or OS and baseline covariates, with results presented as HRs with 95% CIs. Similarly, the HRs and 95% CIs of EFS or OS in each MLL status were estimated, and their interactions were tested. EFS and OS were evaluated by Kaplan-Meier method and compared by log-rank test. All statistical analysis by SPSS statistical software version 22.0 and EmpowerStats (http://www.empowerstats.cn/). *P* < 0.05 was considered statistically significant.

## Results

### Baseline characteristics of pediatric ALL patients

Thirty-one MLL-r positive cases were detected among 637 children with initial ALL, with a positive rate of 4.9% (31/637). Thirty-one MLL-r positive children included 9 positive MLL-PTD (Partial Tandem Duplications), 8 positive MLL-AF4, 5 positive MLL-ENL, 3 positive MLL-AF10, 3 positive MLL-AF9, 2 positive MLL-AF6, and 1 positive MLL-ELL. For statistical convenience, the partner genes were divided into MLL-PTD group, MLL-AF4 group, and MLL-ENL group, and the remaining partner genes were uniformly classified as other groups (Table 1).

**Table 1:** Distribution of MLL Partner Gene

MLL partner gene	Cases, n(%)
MLL-PTD	9(29%)
MLL-AF4	8(25.8%)
MLL-ENL	5(16.1%)
Others	
MLL-AF10	3(9.6%)
MLL-AF6	2(6.5%)
MLL-AF9	3(9.6%)
MLL-ELL	1(3.2%)
Total	31

### Comparison between MLL-r-positive and MLL-r-negative ALL

The differences between the MLL-r positive group and the negative group were not statistically significant (*P* > 0.05) in terms of chemotherapy protocol, gender, Hb and PLT (Table 2). The median age of the 124 children was 3.9 (0.4–14.9) years, including 74 males (59.7%) and 50 females (40.3%).

**Table 2:** Baseline Characteristics of Study Participants by MLL status Classification

Characteristics	Total	MLL status		*P* value
		Negative(*n* = 93)	Positive(*n* = 31)	
Gender, n(%)				0.291
Male	74 (59.7%)	53 (57.0%)	21 (67.7%)	
Female	50 (40.3%)	40 (43.0%)	10 (32.3%)	
Age (y), median(range)	3.9 (0.4–14.9)	4.5 (1.4–14.9)	3.1 (0.4–12.5)	0.019
Age group(y)				0.002
< 1	4 (3.2%)	0 (0.0%)	4 (12.9%)	
≥ 1, < 10	103 (83.1%)	80 (86.0%)	23 (74.2%)	
≥ 10	17 (13.7%)	13 (14.0%)	4 (12.9%)	
Chemotherapy protocol, n(%)				0.706
SCCLG-ALL-2016 Protocol	27 (21.8%)	21 (22.6%)	6 (19.4%)	
GD-ALL-2008 Protocol	97 (78.2%)	72 (77.4%)	25 (80.6%)	
Initial WBC (×10^9^/L),median(range)	8.9 (0.2–895.5)	6.5 (0.2–354.4)	22.6 (3.5–895.5)	< 0.001
WBC group, n(%)				< 0.001
< 50 × 10^9^/L	101 (81.5%)	83 (89.2%)	18 (58.1%)	
≥ 50 × 10^9^/L	23 (18.5%)	10 (10.8%)	13 (41.9%)	
Initial Hb (g/L),median(range)	80.5 (50.0–149.0)	80.0 (50.0–139.0)	82.0 (50.0–149.0)	0.762
Hb group				0.333
< 110 g/L	103 (83.1%)	79 (84.9%)	24 (77.4%)	
≥ 110 g/L	21 (16.9%)	14 (15.1%)	7 (22.6%)	
Initial PLT (×10^9^/L), median(range)	64.5 (6.0–499.0)	70.0 (6.0–499.0)	57.0 (9.0–416.0)	0.619
PLT group				0.273
< 100 × 10^9^/L	82 (66.1%)	59 (63.4%)	23 (74.2%)	
≥ 100,×10^9^/L	42 (33.9%)	34 (36.6%)	8 (25.8%)	
Risk group, n(%)				< 0.001
SR	29 (23.4%)	27 (29.0%)	2 (6.5%)	
IR	53 (42.7%)	44 (47.3%)	9 (29.0%)	
HR	42 (33.9%)	22 (23.7%)	20 (64.5%)	
Immunophenotype, n(%)				< 0.001
Pro-B	7(5.6%)	2(2.2%)	5(16.1%)	
Common-B	75(60.5%)	67(72.0%)	8(25.8%)	
T	13(10.5%)	6(6.5%)	7(22.6%)	
Pre-B	7(5.6%)	4(4.3%)	3(9.7%)	
Immature-B	22(17.8%)	14(15.1%)	8(25.8%)	
Prednisone Response, n(%)				0.377
PGR	106 (85.5%)	81 (87.1%)	25 (80.6%)	
P PR	18 (14.5%)	12 (12.9%)	6 (19.4%)	
D15 BM, n(%)				0.036
M1	100 (80.6%)	79 (84.9%)	21 (67.7%)	
M2/M3	24 (19.4%)	14 (15.1%)	10 (32.3%)	
D33 BM, n(%)				0.736
M1	121 (97.6%)	91 (97.8%)	30 (96.8%)	
M2/M3	3 (2.4%)	2 (2.2%)	1 (3.2%)	
D15 MRD, n(%)				0.294
< 0.1%	45 (40.9%)	40 (43.0%)	5 (29.4%)	
≥ 0.1%	65 (59.1%)	53 (57.0%)	12 (70.6%)	
D33 MRD, n(%)				0.046
< 0.01%	98 (87.5%)	84 (90.3%)	14 (73.7%)	
≥ 0.01%	14 (12.5%)	9 (9.7%)	5 (26.3%)	
SCT, n(%)				0.147
No	118 (95.2%)	90 (96.8%)	28 (90.3%)	
Yes	6 (4.8%)	3 (3.2%)	3 (9.7%)	

*Abbreviation*: *WBC* white blood cell, *Hb* haemoglobin, *PLT* platelet, *CNS L* central nervous system leukemia, *SR* standard risk, *IR* intermediate risk, *HR* high risk, *PGR* prednisone good response, *PPR* prednisone poor response, *MRD* minimal residual disease evaluation, *BM* bone marrow, *SCT* stem cell transplantation

By comparing the MLL-r positive and negative groups, the median age of onset was 3.1(0.4–12.5) and 4.5(1.4–14.9) years, respectively, with statistically significant differences between the two groups(*P* = 0.019). The MLL-r positive group were mostly seen in common-B, immature-B and T, when compared with the negative group (*P* < 0.001). The proportion of patients with risk factors (age < 1 year, WBC ≥50 × 10^9^/L) at initial diagnosis was significantly higher in the MLL-r positive group than in the negative group (12.9% versus 0, *P* = 0.002; 41.9% versus 10.8%, *P* < 0.001). In the final clinical risk group, a significantly higher proportion of MLL-r positive cases was included in the high-risk group than in the negative group (64.5% versus 23.7%, *P* < 0.001).

Further comparing the response of PR between MLL-r positive and negative groups (Table 2), the proportion of PGR in MLL-r positive group was lower than that in negative group, but there was no significant difference (80.6% versus 87.1%, *P* = 0.377). When the BM was detected on Day 15 of induction chemotherapy, the proportion of M1 in the MLL-r positive group was significantly lower than that in the negative group (67.7% versus 84.9%, *P* = 0.036), while the BM was monitored on Day 33 of induction chemotherapy, the M1 in the MLL-r positive group was not significantly different from that in the negative group (96.8% versus 97.8%, *P* = 0.736). A total of 110 children were tested for D15 MRD in this study, and 65 (59.1%) were positive for D15 MRD, with a higher proportion of positive D15 MRD in the MLL-r positive group than in the MLL-r negative group (70.6% versus 57%, *P* = 0.294). Finally, 112 children were tested for D33 MRD, and 14 (12.5%) were positive for D33 MRD, with a higher proportion of positive D33 MRD in the MLL-r positive group than in the MLL-r negative group. The CR rate on Day 33 was 96.8% (30/31) in the MLL-r positive group and 97.8% (91/93) in the MLL-r negative group, with no statistical difference between the two CR rates (*P* = 0.736).

### Prognostic significance of the overall cohort among pediatric ALL

Univariate analysis of risk factors that had statistically significant effects on the whole cohort with ALL were selected for inclusion in the multivariate analysis, and the results showed that WBC ≥50 × 10^9^/L (EFS: *HR* = 2.7, 95%*CI*(1.1–7.1), *P* = 0.039; OS: *HR* = 4.4, 95%*CI*(1.1–19.6), *P* = 0.048) and MLL-AF4 (EFS: *HR* = 7.5, 95%*CI* (1.6–35.5), *P* = 0.012; OS: *HR* = 7.8, 95%*CI*(1.1–53.1), *P* = 0.036) were both independent risk factors for the prognosis of patients with ALL. In contrast, MLL-r positive was not an independent prognostic risk factor (EFS: *HR* = 1.6, 95%*CI*(0.6–4.5), *P* = 0.343; OS: *HR* = 1.4, 95% *CI* (0.4–5.1), *P* = 0.608).

### Subgroup analysis of prognostic significance for MLL-r positive patients and survival analysis

Analysis of possible risk factors affecting EFS in MLL-r positive children by applying a columnar chi-square test showed that gender, WBC, immunophenotype, MLL partner gene type, D15 MRD and SCT were all associated factors affecting EFS and OS in MLL-r positive patients (*P* values < 0.05) (Table 3). Interaction tests were done between MLL status and EFS or OS (Supplementary Table 1 and Supplementary Table 2).

**Table 3:** Univariate analysis for EFS and OS among MLL-r positive ALL patients

Variables	EFS		OS	
	HR (95%CI)	*P value*	HR (95%CI)	*P value*
Gender				
Male	Ref.		Ref.	
Female	5.0 (1.5, 17.5)	0.011	3.8 (1.2, 12.5)	0.025
Age group				
< 1	Ref.		Ref.	
≥ 1, < 10	0.3 (0.1, 1.1)	0.072	0.3 (0.1, 1.3)	0.101
≥ 10	0.1 (0.0, 0.8)	0.028	0.2 (0.0, 2.2)	0.183
Chemotherapy protocol				
GD-ALL-2008 Protocol	Ref.		Ref.	
SCCLG-ALL-2016 Protocol	1.2 (0.4, 3.1)	0.745	1.4 (0.1, 17.6)	0.771
WBC group				
< 50 × 10^9^/L	Ref.		Ref.	
≥ 50 × 10^9^/L	2.7 (1.1, 7.1)	0.039	16.9 (1.5, 185.3)	0.021
Hb group				
< 110 g/L	Ref.		Ref.	
≥ 110 g/L	0.8 (0.2, 3.8)	0.797	1.5 (0.6, 4.0)	0.425
PLT group				
< 100 × 10^9^/L	Ref.		Ref.	
≥ 100 × 10^9^/L	0.2 (0.0, 1.9)	0.175	0.6 (0.2, 1.5)	0.260
Risk group				
SR	Ref.		Ref.	
IR	1.6 (0.3, 7.5)	0.576	4.9 (0.5, 47.5)	0.170
HR	1.8 (0.5, 6.8)	0.399	1.7 (0.1, 26.8)	0.717
Immunophenotype				
B -cell	Ref.		Ref.	
T-cell	6.7 (1.5, 29.6)	0.012	2.8 (1.1, 7.1)	0.031
MLL partner gene				
MLL-PTD	Ref.		Ref.	
MLL-AF4	4.8 (1.4, 17.1)	0.015	6.4 (1.6, 24.8)	0.007
MLL-ENL	1.2 (0.2, 7.0)	0.872	1.7 (0.1, 26.8)	0.717
Others	0.9 (0.1, 5.3)	0.882	1.2 (0.1, 19.9)	0.883
Prednisone Response				
PGR	Ref.		Ref.	
P PR	1.8 (0.5, 6.7)	0.399	2.0 (0.6, 7.4)	0.285
D15 BM				
M1	Ref.		Ref.	
M2/M3	1.4 (0.4, 4.7)	0.633	2.0 (0.6, 6.6)	0.243
D33 BM				
M1	Ref.		Ref.	
M2/M3	1.0 (0.2, 4.2)	0.960	0.5 (0.0, 9.9)	0.683
D15 MRD				
< 0.1%	Ref.		Ref.	
≥ 0.1%	3.6 (1.1, 12.4)	0.041	5.8 (1.2, 29.0)	0.031
D33 MRD				
< 0.01%	Ref.		Ref.	
≥ 0.01%	0.5 (0.1, 3.8)	0.498	2.1 (0.4, 10.1)	0.360
SCT				
No	Ref.		Ref.	
Yes	0.4(0.2, 0.9)	0.027	0.1 (0.0, 0.8)	0.027

*Abbreviation*: *WBC* white blood cell, *Hb* haemoglobin, *PLT* platelet, *CNS L* central nervous system leukemia, *SR* standard risk, *IR* intermediate risk, *HR* high risk, *PGR* prednisone good response, *PPR* prednisone poor response, *MRD* minimal residual disease evaluation, *BM* bone marrow, *SCT* stem cell transplantation

Cox model multivariate regression analysis showed that T-cell phenotype (EFS: *HR* = 5.0, 95%*CI*(1.5–17.5), *P* = 0.011; OS: *HR* = 6.7, 95%*CI* (1.5–29.6), *P* = 0.012), WBC ≥50 × 10^9^/L(EFS: *HR* = 6.5, 95%*CI*(1.6–24.8), *P* = 0.007; OS: *HR* = 3.6, 95%*CI* (1.1–12.4), *P* = 0.041), MLL-AF4 (EFS: *HR* = 3.5, 95%*CI*(1.1–11.5), *P* = 0.035; OS: *HR* = 4.6, 95%*CI* (1.5–14.4), *P* = 0.008), and D15 MRD positive (EFS: *HR* = 4.8, 95%*CI*(1.4–17.1), *P* = 0.015; OS: *HR* = 2.6, 95%*CI*(1.1–6.0), *P* = 0.025) were the independent risk factors affecting the outcome of MLL-r positive patients. Interestingly, SCT was a favorable independent factor on EFS(*HR* = 0.4, 95%*CI* (0.0–0.9), *P* = 0.027), and tended to have an independent effect on OS (*HR* = 0.2, 95%*CI:* 0.0–1.1, *P* = 0.065) (Table 4).

**Table 4:** Multivariate analysis for EFS and OS among MLL-r positive ALL patients

Outcome	Variable	HR (95% CI)	*P* value
EFS	T-cell	5.0 (1.5, 17.5)	0.011
	WBC ≥50 × 10^9^/L	6.4 (1.6, 24.8)	0.007
	MLL-AF4	3.5 (1.1, 11.5)	0.035
	MLL-PTD	2.4 (0.6, 9.9)	0.223
	MLL-ENL	1.9 (0.2, 15.4)	0.554
	MLL-others	1.5 (0.2, 12.6)	0.682
	PPR	6.1 (0.2, 212.8)	0.321
	D15 BM NR	3.6 (0.5, 26.3)	0.214
	D33 BM NR	1.4 (0.3, 6.3)	0.653
	D15 MRD (+)	4.8 (1.4, 17.1)	0.015
	D33 MRD (+)	0.1 (0.0, 353.7)	0.596
	SCT	0.4(0.0, 0.9)	0.027
OS	T-cell	6.7 (1.5–29.6)	0.012
	WBC ≥50 × 10^9^/L	3.6 (1.1, 12.4)	0.041
	MLL-AF4	4.6 (1.5, 14.4)	0.008
	MLL-PTD	4.5 (0.3, 57.0)	0.251
	MLL-ENL	0.9 (0.7, 1.3)	0.700
	MLL-others	2.6 (0.8, 8.3)	0.115
	PPR	6.9 (0.1, 616.6)	0.402
	D15 BM NR	0.3 (0.1, 1.6)	0.180
	D33 BM NR	6.9 (0.1, 616.6)	0.402
	D15 MRD (+)	2.6 (1.1, 6.0)	0.025
	D33 MRD (+)	3.1 (0.2, 40.8)	0.384
	SCT	0.2 (0.0, 1.1)	0.065

*Abbreviation*: *WBC* white blood cell, *Hb* haemoglobin, *PLT* platelet, *CNS L* central nervous system leukemia, *SR* standard risk, *IR* intermediate risk, *HR* high risk, *PGR* prednisone good response, *PPR* prednisone poor response, *MRD* minimal residual disease evaluation, *BM* bone marrow, *SCT* stem cell transplantation

**Fig. 1:**
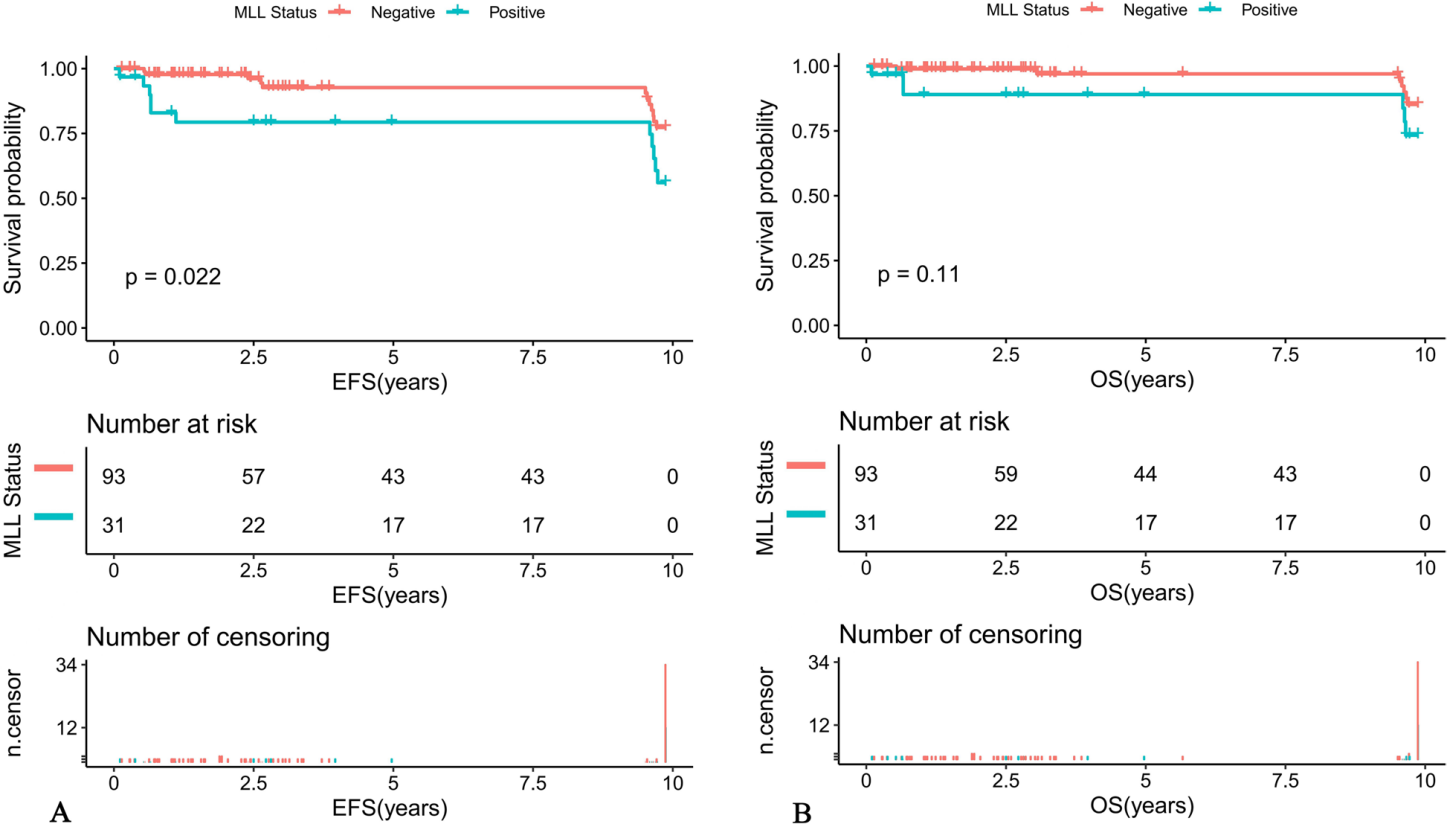
Survival cures of pediatric ALL according to different MLL status. **A** The 10-year EFS of MLL-r positive and MLL-r negative ALL patients. **B** The 10-year OS of MLL-r positive and MLL-r negative ALL patients

**Fig. 2:**
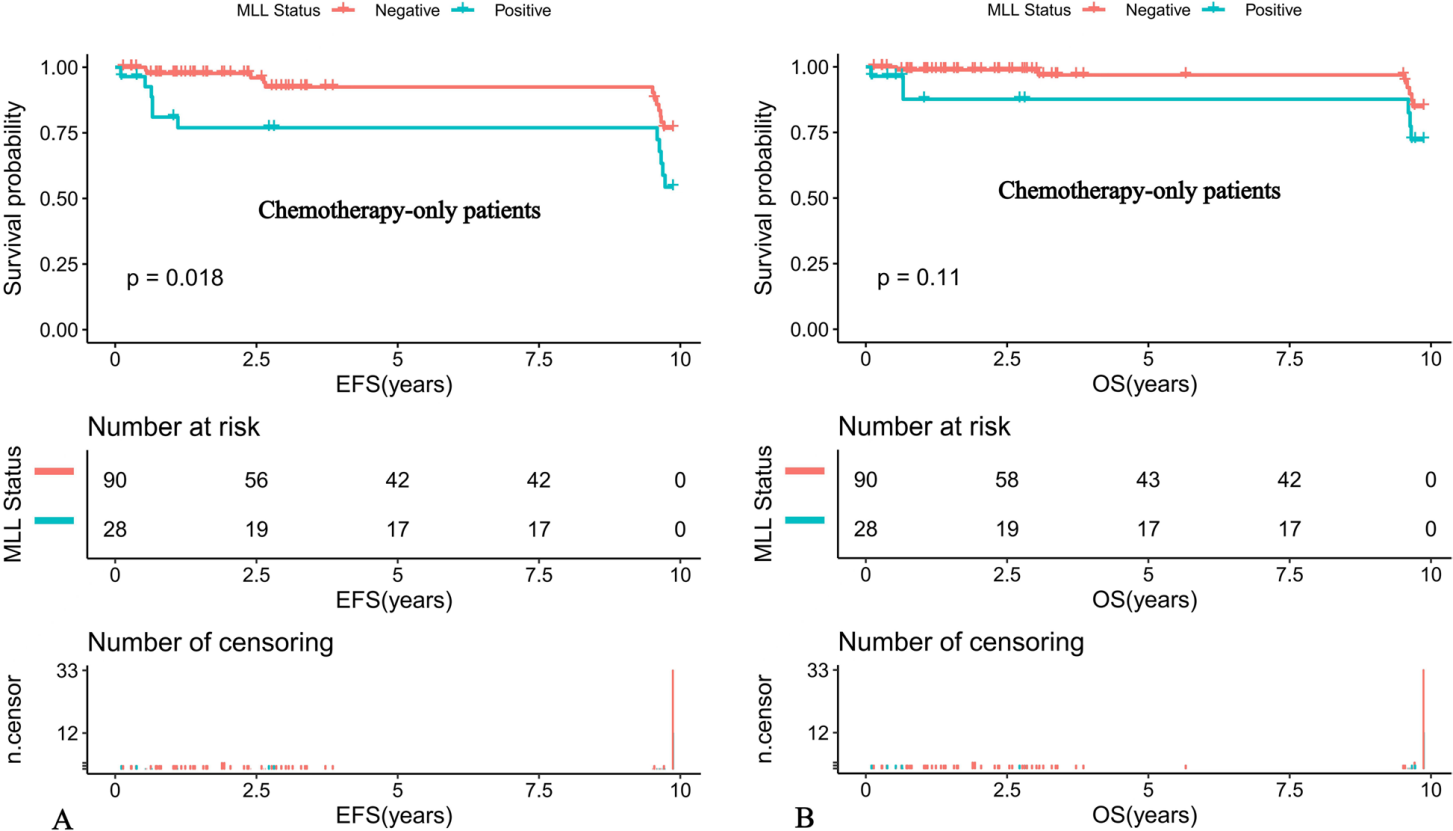
Survival cures of pediatric ALL with chemotherapy-only according to different MLL status. **A** The 10-year EFS of pediatric ALL with chemotherapy-only according to different MLL status. **B** The 10-year OS of pediatric ALL with chemotherapy-only according to different MLL status

**Fig. 3:**
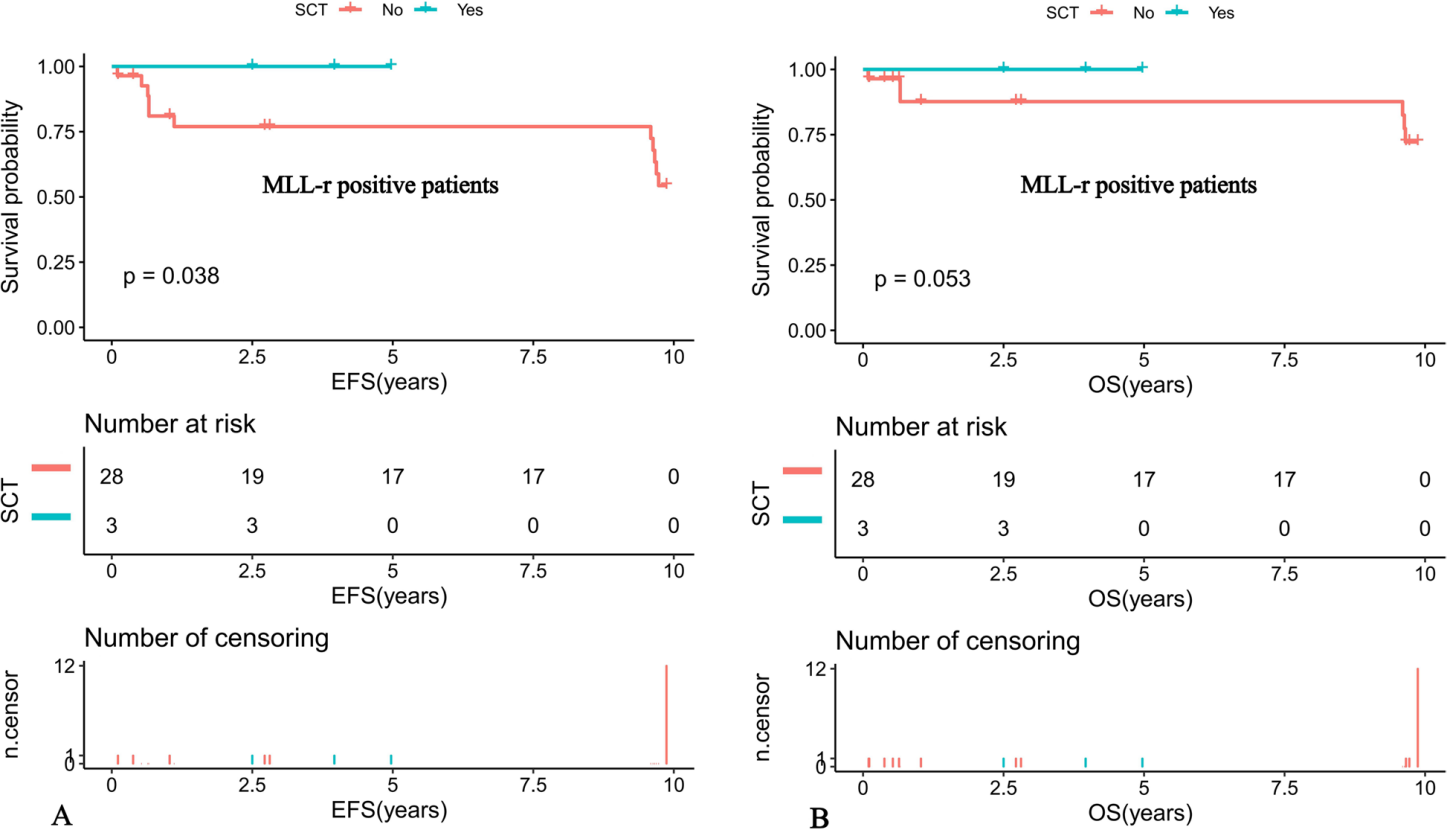
Survival curves of MLL-r positive pediatric ALL patients according to SCT status. **A** Probability of EFS for MLL-r positive patients according to SCT status. **B** Probability of OS for MLL-r positive patients according to SCT status

Further comparison of the K-M survival curves of the 31 MLL-r positive and 93 MLL-r negative patients on standardized treatment showed that the 10-year predicted EFS rate was significantly lower in the MLL-r positive than in the negative group (EFS: 56.01 ± 16.89% versus 77.33 ± 10.81%, *P* = 0.022) (Fig. 1A). While the 10-year predicted OS rate in the positive group had a tendency to be lower in the positive group than in the negative group (OS: 73.32 ± 16.6% versus 85.2 ± 9.77%, *P* = 0.11) (Fig. 1B). Moreover, our K-M survival analysis of children with ALL receiving chemotherapy-only showed that the 10-year predicted EFS rate was significantly lower in MLL-r positive cases receiving chemotherapy-only than in the negative group (54.32 ± 16.89% versus 76.78 ± 11.01%, *P* = 0.018) (Fig. 2A), and the 10-year predicted OS rate also tended to be lower in the positive group than in the negative group (72.19 ± 16.88% versus 84.84 ± 9.97%, *P* = 0.11) (Fig. 2B). Among the MLL-r positive cases, the 10-year predicted EFS rate was significantly higher in those who received SCT than in those who received chemotherapy-alone (100% versus 54.32 ± 16.89%, *P* = 0.038) (Fig. 3A). While the 10-year predicted OS rate in children who received SCT showed a trend to be higher than in those who received chemotherapy-only (100% versus 72.19 ± 16.88%, *P* = 0.053) (Fig. 3B).

**Fig. 4:**
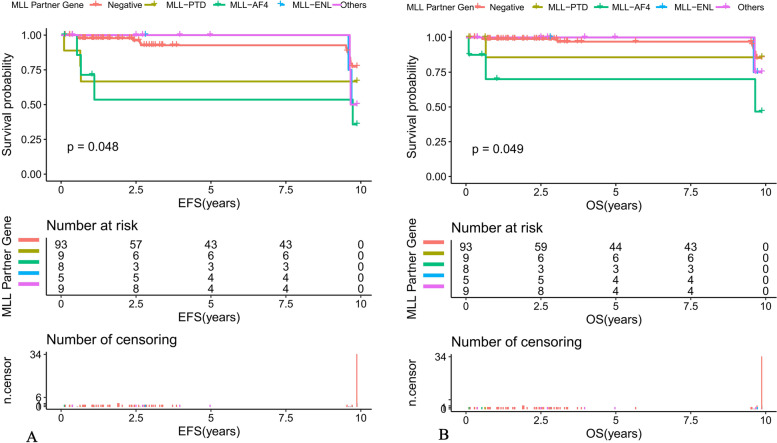
Survival curves of different MLL partner gene among pediatric ALL patients (**A**) The 10-year EFS of different MLL partner gene among ALL patients. **B** The 10-year OS of different MLL partner gene among ALL patients

Among patients with ALL, the 10-year predicted EFS for MLL-AF4 positive, MLL-PTD positive, MLL-ENL positive, other MLL gene partner positive and MLL-r negative children were 46.67 ± 28.61%, 85.71 ± 22.37%, 75 ± 32.41%, 75 ± 32.41% and 77.33 ± 10.81%, respectively (*P* = 0.048) (Fig. 4A). The 10-year predicted OS was 46.67 ± 28.61%, 85.71 ± 22.37%, 75 ± 32.41%, 75 ± 32.41%, and 85.2 ± 9.77%, respectively (*P* = 0.049) (Fig. 4B).

### Relapse analysis

Long-term follow-up of 124 children with ALL resulted in relapse in 5 (4%) children with a median relapse time of 3.9 (0.1–9.9) years. The median time to relapse was 3.4 years in the MLL-r negative group and 9.6 (0.1–9.9) years in the MLL-r-positive group, with no statistical difference between the two groups. There was no statistical difference between the two relapse times (*P* = 0.502). The four children with MLL-r positive relapse were on the GD-ALL 2008 protocol and the partner genotypes included one MLL-AF4, one MLL-PTD, and two MLL-ENL, all with solo BM relapse and high risk factors for poor prognosis such as WBC ≥50 × 10^9^/L, T-cell phenotype, or D15 MRD positive (Table 5).

**Table 5:** Basic Information of MLL-r Positive ALL Relapse Cases

No.	Age/Gender	WBC×10^9^/L	PR	Immunophenotype	D15 BM	D15 MRD	D33 BM	D33 MRD	Risk group	Partner gene
1	5 Month/Male	895.54	PGR	B	M3	Positive	M1	Positive	HR	MLL-ENL
2	11 Month/Female	3.87	PGR	B	M1	Negative	M1	Negative	IR	MLL-ENL
3	2 Year/Female	169.95	PPR	B	M1	Positive	M1	Negative	HR	MLL-AF4
4	2 Year/Female	218	PGR	T	M1	Positive	M1	Positive	HR	MLL-PTD

*Abbreviation*: *WBC* white blood cell, *SR* standard risk, *IR* intermediate risk, *HR* high risk, *PR* prednisone response, *PGR* prednisone good response, *PPR* prednisone poor response, *MRD* minimal residual disease evaluation, *BM* bone marrow

## Discussion

Previous literature reported that MLL-r positive ALL can develop among children at all ages, accounting for 2.5–5% of children with initial ALL [2, 3], and the detection rate of MLL-r positive ALL in infants younger than 1 year of age is even as high as 23.8–79% [9–11]. The detection rate of MLL-r positive ALL patients in this study was 4.9%, which was basically consistent with the literature. However, the detection rate in infants younger than 1 year of age was only 3.2%, which was much lower than the above mentioned literature, and the reason for this might be related to the sample size of MLL-r positive patients included in different studies.

Compared with the MLL-r negative group of patients, the clinical characteristics of MLL-r positive cases in this study included: a young age of onset, with a median age of onset of 3.1 years, and 12.9% of infant leukemia, which was much higher than that of the MLL-r negative group. In addition, a high WBC of onset, with a significantly higher proportion of initial WBC greater than 50 × 10^9^/L than that of the negative group and a high percentage of 64.5% in the high-risk group, which were consistent with previous studies. The MLL-r, as one of the subgroups of ALL, may be associated with certain immunophenotypic characteristics. Moorman et al [12] showed that MLL-r positive ALL was mostly of B-cell lineage and common B or pro-B immunophenotypes were more common, accounting for 62% of the cases. Peterson et al [13] retrospectively found that in 806 children with T-lineage ALL, 27 (3.3%) MLL-r positive cases were detected. In contrast, our findings showed that the immunophenotype of MLL-r positive children was more prevalent in common-B, immature B and T cells compared to the MLL-r negative group, which was basically consistent with Moorman and Peterson’s report. In China, it has been reported that the immunophenotype pro-B is a risk prognostic factor in MLL-r positive cases [14]. In contrast, the results of the multivariate analysis in this study showed that the T-lineage immunophenotype was an independent prognostic factor affecting MLL-r positive ALL, which was inconsistent with the above-mentioned studies and we implied that it might be related to the small number of reported cases in China (only 6 cases).

Some studies [10, 11, 14, 15] reported that MLL-r positive compared to MLL-r negative children had a higher proportion of patients with poor early treatment response in addition to more risk factors at initial diagnosis. In the current study, we also found that the proportion of M1 patients on Day 15 of induction chemotherapy in MLL-r positive children with ALL was significantly lower than that in the negative group, and the proportion of MRD positive patients on Day 33 was significantly higher than that in the negative group, suggesting that the poor early treatment response in MLL-r positive patients may be due to the dominant clone of MLL-r positive leukemia cells being insensitive or resistant to chemotherapy at the time of initial diagnosis, rather than chemotherapy “selected” for the inferior clone [16, 17]. However, after one course of standardized induction chemotherapy, the percentage of MLL-r positive patients with CR of BM could reach 96.8%, which is similar to the 97.6% CR rate reported in China [10], which might be related to the early strong chemotherapy.

In this present study, only 13 of 31 MLL-r positive ALL cases were successfully detected for BM karyotype, and 8 cases were normal karyotype, and the CCA compliance rate was only 38.5%, which may be due to the fact that most of the chromosomes in MLL-r positive children belong to normal. On the other hand, it may also be due to the small 11q23 broken fragment and the influence of the quality of the split phase and the cryptic chromosomal translocation, so there was a missed detection [11]. FISH technique can detect basically all cases with MLL-r positive and had higher resolution, stronger sensitivity and shorter cycle time, but it cannot detect MLL partner genes and MLL-PTD due to the limitation of probes. Combined with PCR method, our department can detect MLL-AF4, MLL-AF6, MLL-AF9, MLL- AF10, MLL-ELL, MLL-ENL, MLL-AF1q, MLL-AF17, and MLL-PTD, which are common MLL-associated fusion genes, thus compensating for the inadequacy of FISH methods [18]. Thus, we suggested the clinical use of a combination of the three above-mentioned assays, which will help to improve the detection rate of MLL-r positive patients.

Meyer et al. [1] analyzed 1420 MLL-r positive children with ALL, and the most common partner genes were MLL-AF4 (57%), MLL-ENL (18%), MLL-AF9 (13%), MLL-AF10 (4%), MLL-EPS15 (2%), and MLL-AF6 (2%). In China, the statistical results of Sun et al [19] on 57 cases of 11q/23 MLL-r pediatric patients with ALL showed that the MLL-AF4 accounted for 29.8% and MLL-PTD accounted for 26.3%, followed by MLL-AF9 (22.8%), MLL-ENL (12.3%) and MLL-AF10 (8.8%), respectively. In our study, MLL-PTD (29%), MLL-AF4 (25.8%), and MLL-ENL (16.1%) were predominant among the 31 MLL-r positive cases, which were consistent with that reported by Sun Yulan et al. and different from that reported by Meyer et al. We speculated that it might be due to the racial differences. Although the detection rate of MLL-r positive ALL children was inconsistent among different studies, the most common partner gene was still MLL-AF4. Some clinical reports [1] showed that MLL partner genes were the main determinants of leukemia phenotype, and MLL-AF4 was mainly associated with lymphoid malignancies, while MLL-AF9 was more likely to cause myeloid malignancies, which might may explain why MLL-AF4 was most common in ALL.

It has been reported in the literature [7, 20] that WBC and MLL-r positive at disease onset were important factors affecting the prognosis of children with ALL, and the results of the multivariate analysis of 124 children with ALL in our study also showed that WBC ≥50 × 10^9^/L was an independent risk factor affecting the outcome of patients with ALL, which was consistent with the literature. However, our study also observed that MLL-r positive was not a prognostic factor for patients with ALL, but its partner gene MLL-AF4 was an independent risk factor for the prognosis of children with ALL, suggesting that the prognosis of our children with ALL may be closely related to the MLL-r partner gene type.

There was a consensus that the overall prognosis of MLL gene rearrangement leukemia is poor, and a large pediatric leukemia collaborative group [8, 20] has shown that the 5-year EFS and OS of MLL-r positive children with ALL were 60–65% and 68–74%, respectively, while our K-M survival analysis showed that the EFS of MLL-r positive children were 56.01 ± 16.89% and OS were 73.32 ± 16.6%, which were significantly lower than the MLL-r negative group and basically close to those reported by the collaborative group, and both were lower than the 5-year EFS (72–80%) and OS (83–85%) levels in domestic patients with ALL, which laterally confirmed that concomitant MLL gene rearrangement positive was a more malignant type of childhood ALL. Therefore, this study further analyzed the prognostic factors affecting MLL-r positive ALL, and the results of Cox model multivariate analysis showed that T-cell phenotype, WBC ≥50 × 10^9^/L and D15 MRD positive were independent risk factors affecting MLL-r positive children, which were consistent with the findings of Tomizawa et al [21] With the increasing maturity of conventional chemotherapy, most children with MLL-r ALL were able to achieve CR with treatment, and the mainstream treatment regimen was still chemotherapy. Our survival analysis of patients with ALL who received only chemotherapy showed that the 10-year EFS and OS of MLL-r positive children who received only chemotherapy were lower than those of the negative group, indicating that although children could enter remission with conventional chemotherapy, some children still experienced relapse after remission, resulting in a significant decrease in EFS, and although the relapse rate of children with MLL-r associated leukemia could be reduced by increasing the intensity of chemotherapy, the treatment-related relapse rate was significantly lower. Although the relapse rate of children with MLL-r associated leukemia could be reduced by increasing the intensity of chemotherapy, the OS due to treatment-related mortality and infection-based complications decreased accordingly. This suggested that chemotherapy-only may be less effective in MLL-r positive cases.

The role of SCT in the treatment of MLL-r-positive leukemia has been controversial. The results showed that the 5-year EFS of 53 children treated with SCT and 47 children treated with chemotherapy alone were 48.8 and 48.7%, respectively, with no statistical difference (*P* = 0.6), indicating that SCT for MLL-r-positive ALL did not show any advantage. ALL did not show any advantage [22]. In contrast, a recent study by the Japanese Pediatric Leukemia/Lymphoma Collaborative Group [23] of 43 MLL-r-positive high-risk children (age < 6 months and/or CNS leukemia) who received SCT showed a 3-year EFS and OS of 56.8 and 80.2%, respectively, demonstrating a good prognosis, suggesting that SCT has more therapeutic advantages over conventional chemotherapy. The 10-year EFS rate and OS of children who received SCT in this study were 100%, which were higher than those who received chemotherapy alone at 54.32 ± 16.89% and 72.19 ± 16.88%, respectively, and SCT seemed to be a good independent prognostic factor affecting MLL-r-positive children. However, due to the small sample size, we cannot conclude that SCT can effectively treat MLL-r positive patients.

It has been suggested that alterations in MLL-r play an important role in the activation of oncogenes, while the role of partner genes fused to them is unclear, while some studies revealed that ALL with MLL-r positive were similar in most morphological and histochemical features, and childhood ALL with MLL-r positive, regardless of the type of partner gene, had an extremely poor prognosis [24]. Previous studies have shown that MLL-AF4 fusions in ALL were associated with poorer survival [25]. A large (*n* = 756), multicenter, retrospective Nordic study analyzing the prognosis of various types of MLL fusion gene leukemia showed that MLL-AF4 and MLL-AF6 had a very poor prognosis with 10-year EFS of 29 and 11% and 10-year OS of 27 and 22%, respectively, while MLL-AF9 had 10-year EFS and OS of 50 and 63%, with a relatively good prognosis [26]. Previous studies [10, 11, 19] had also shown that MLL-AF4 had a worse prognosis than non-MLL-AF4 partner genes, and the above findings strongly suggested that different partner genes had distinct effects on the prognosis of patients with MLL-r positive leukemia. In the present study, we found that partner genes such as MLL-AF4, MLL-PTD and MLL-ENL were more common in MLL-r positive ALL, and further comparison of EFS and OS among the three groups showed statistically significant differences, indicating that MLL-AF4 positive ALL had the worst prognosis, and the results also indicated that the MLL fusion gene type (MLL-AF4) as an independent risk prognostic factor. Inconsistent with other reports in the literature, the most reported partner gene in our study was MLL-PTD, which had a 10-year EFS and OS of 85.71 ± 22.37%, showing a good prognosis, suggesting that MLL-PTD may be an indicator of relatively good prognosis, but most of the literature has not analyzed MLL-PTD, and the limited number of cases included in this study was not enough to reveal this, and the overall prognosis of children in this group deserves our further attention. We conducted long-term follow-up of 124 children with ALL included in this study, and eventually 5 children relapsed, with a significantly higher proportion of MLL-r positive children relapsing than in the negative group, and all of them relapsed in bone marrow alone and had high-risk factors for poor prognosis such as WBC ≥ 50 × 10^9^/L, T-cell phenotype or positive D15 MRD, consistent with the literature [10–15].

Based on these findings, we believe that MLL-r positive should not be uniformly classified as a high-risk group in clinical practice, but that screening for these MLL partner genes is needed for accurate risk stratification at diagnosis: those positive for MLL-AF4 can be treated as a high-risk group, while other MLL partner genes need to be specifically combined with other prognostic factors (T-cell phenotype, WBC ≥ 50 × 10^9^/L and D15 MRD positive) for a comprehensive evaluation. Moreover, it seemed that the relapse time for the MLL-r positive group is longer than MLL-r negative group (9.6 vs 3.9). We implied that the reason for the late relapse of MLL-r positive children was that the combination of drug chemotherapy will not kill the MLL gene clone formed in the fetal period of ALL children. The clone will undergo secondary transformation after treatment, which will eventually lead to the late relapse of ALL [12–15].

In conclusion, the remission rate of MLL-r positive ALL in children was moderate, but prone to relapse with low overall survival, and poor prognosis for those treated with chemotherapy-only. The prognosis of children with MLL-r positive ALL were closely related to the type of MLL-r, and clinical attention should be paid to screening for MLL partner genes and combining them with other prognostic factors for accurate risk stratification.

## Supplementary Information

**Additional file 1: Supplementary Table 1.** Interaction terms tested between MLL status and EFS.

**Additional file 2: Supplementary Table 2.** Interaction terms tested between MLL status and OS.

## Data Availability

The datasets generated and/or analysed during the current study are not publicly available due personal privacy but are available from the corresponding author on reasonable request.
