# Supplementary material for: Prognostic value and outcome for acute lymphocytic leukemia in children with MLL rearrangement: a case-control study
Source: BMC Cancer. 2022 Dec 2;22:1257. doi: 10.1186/s12885-022-10378-w (PMC9719147; doi:10.1186/s12885-022-10378-w)
Supplement: Supplementary file 1 — Additional file 1: Supplementary Table 1. Interaction terms tested between MLL status and EFS. [file 12885_2022_10378_MOESM1_ESM.docx]

**Supplementary Table 1** Interaction terms tested between MLL status and EFS

| Exposure | MLL Status | N | Crude |
| --- | --- | --- | --- |
| Chemotherapy protocol |  |  |  |
| GD-ALL-2008 Protocol | Negative | 72 | Ref. |
| SCCLG-ALL-2016 Protocol | Negative | 21 | 0.0 (0.0, Inf) 0.9974 |
| GD-ALL-2008 Protocol | Positive | 25 | 2.5 (1.1, 5.7) 0.0268 |
| SCCLG-ALL-2016 Protocol | Positive | 6 | 0.0 (0.0, Inf) 0.9985 |
| P interaction |  |  | 1.0000 |
| Risk group |  |  |  |
| SR | Negative | 27 | Ref. |
| IR | Negative | 44 | 1.7 (0.3, 8.4) 0.5232 |
| HR | Negative | 22 | 3.6 (0.6, 19.5) 0.1435 |
| SR | Positive | 2 | 0.0 (0.0, Inf) 0.9976 |
| IR | Positive | 9 | 0.8 (0.1, 9.1) 0.8730 |
| HR | Positive | 20 | 9.0 (2.0, 41.4) 0.0046 |
| P interaction |  |  | 0.2697 |
| Immunophenotype |  |  |  |
| B | Negative | 86 | Ref. |
| T | Negative | 7 | 1.0 (0.1, 7.7) 0.9987 |
| B | Positive | 24 | 2.2 (0.9, 5.4) 0.0952 |
| T | Positive | 7 | 4.3 (1.2, 15.4) 0.0253 |
| P interaction |  |  | 0.5698 |
| Prednisone Response |  |  |  |
| Good | Negative | 81 | Ref. |
| Poor | Negative | 12 | 1.4 (0.3, 6.3) 0.6770 |
| Good | Positive | 25 | 2.3 (0.9, 5.9) 0.0771 |
| Poor | Positive | 6 | 4.2 (1.2, 15.3) 0.0295 |
| P interaction |  |  | 0.7909 |
| D15 BM |  |  |  |
| M1 | Negative | 79 | Ref. |
| M2/M3 | Negative | 14 | 0.5 (0.1, 4.2) 0.5602 |
| M1 | Positive | 21 | 2.1 (0.8, 5.5) 0.1217 |
| M2/M3 | Positive | 10 | 2.9 (0.9, 9.2) 0.0667 |
| P interaction |  |  | 0.4185 |
| D15 MRD |  |  |  |
| <0.1% | Negative | 40 | Ref. |
| ≥0.1% | Negative | 53 | 0.9 (0.3, 2.9) 0.8679 |
| <0.1% | Positive | 5 | 1.6 (0.2, 14.1) 0.6509 |
| ≥0.1% | Positive | 12 | 2.8 (0.8, 9.6) 0.1083 |
| P interaction |  |  | 0.6047 |
| D33 BM |  |  |  |
| M1 | Negative | 91 | Ref. |
| M2/M3 | Negative | 2 | 3.3 (0.4, 25.4) 0.2587 |
| M1 | Positive | 29 | 2.5 (1.0, 5.8) 0.0392 |
| M2/M3 | Positive | 1 | 0.0 (0.0, Inf) 0.9981 |
| P interaction |  |  | 0.3457 |
| D33 MRD |  |  |  |
| <0.01% | Negative | 84 | Ref. |
| ≥0.01% | Negative | 9 | 0.9 (0.1, 7.3) 0.9507 |
| <0.01% | Positive | 14 | 1.8 (0.6, 5.8) 0.2962 |
| ≥0.01% | Positive | 5 | 1.3 (0.2, 10.2) 0.7922 |
| P interaction |  |  | 0.8595 |
| SCT |  |  |  |
| No | Negative | 90 | Ref. |
| Yes | Negative | 3 | 0.0 (0.0, Inf) 0.9982 |
| No | Positive | 28 | 2.6 (1.1, 5.9) 0.0233 |
| Yes | Positive | 3 | 0.0 (0.0, Inf) 0.9986 |
| P interaction |  |  | 0.9999 |
