# Supplementary material for: Prognostic value and outcome for acute lymphocytic leukemia in children with MLL rearrangement: a case-control study
Source: BMC Cancer. 2022 Dec 2;22:1257. doi: 10.1186/s12885-022-10378-w (PMC9719147; doi:10.1186/s12885-022-10378-w)
Supplement: Supplementary file 2 — Additional file 2: Supplementary Table 2. Interaction terms tested between MLL status and OS. [file 12885_2022_10378_MOESM2_ESM.docx]

**Supplementary Table 2** Interaction terms tested between MLL status and OS

| Exposure | MLL Status | N | Crude |
| --- | --- | --- | --- |
| Chemotherapy protocol |  |  |  |
| GD-ALL-2008 Protocol | Negative | 72 | Ref. |
| SCCLG-ALL-2016 Protocol | Negative | 21 | 0.0 (0.0, Inf) 0.9981 |
| GD-ALL-2008 Protocol | Positive | 25 | 2.3 (0.8, 6.9) 0.1294 |
| SCCLG-ALL-2016 Protocol | Positive | 6 | 0.0 (0.0, Inf) 0.9990 |
| P interaction |  |  | 0.9999 |
| Risk group |  |  |  |
| SR | Negative | 27 | Ref. |
| IR | Negative | 44 | inf. (0.0, Inf) 0.9986 |
| HR | Negative | 22 | inf. (0.0, Inf) 0.9986 |
| SR | Positive | 2 | 1.0 (0.0, Inf) 1.0000 |
| IR | Positive | 9 | 1.0 (0.0, Inf) 1.0000 |
| HR | Positive | 20 | inf. (0.0, Inf) 0.9985 |
| P interaction |  |  | 0.0793 |
| Immunophenotype |  |  |  |
| B | Negative | 86 | Ref. |
| T | Negative | 7 | 1.9 (0.2, 16.0) 0.5429 |
| B | Positive | 24 | 2.5 (0.8, 8.2) 0.1330 |
| T | Positive | 7 | 2.7 (0.3, 22.7) 0.3533 |
| P interaction |  |  | 0.7139 |
| Prednisone Response |  |  |  |
| Good | Negative | 81 | Ref. |
| Poor | Negative | 12 | 1.2 (0.1, 10.2) 0.8483 |
| Good | Positive | 25 | 1.9 (0.5, 6.8) 0.3182 |
| Poor | Positive | 6 | 5.2 (1.0, 25.8) 0.0452 |
| P interaction |  |  | 0.5598 |
| D15 BM |  |  |  |
| M1 | Negative | 79 | Ref. |
| M2/M3 | Negative | 14 | 1.0 (0.1, 8.5) 0.9872 |
| M1 | Positive | 21 | 1.7 (0.4, 6.7) 0.4761 |
| M2/M3 | Positive | 10 | 4.1 (1.0, 16.3) 0.0478 |
| P interaction |  |  | 0.5004 |
| D15 MRD |  |  |  |
| <0.1% | Negative | 40 | Ref. |
| ≥0.1% | Negative | 53 | 1.7 (0.3, 8.6) 0.5458 |
| <0.1% | Positive | 5 | 0.0 (0.0, Inf) 0.9985 |
| ≥0.1% | Positive | 12 | 4.3 (0.7, 25.8) 0.1121 |
| P interaction |  |  | 0.3156 |
| D33 BM |  |  |  |
| M1 | Negative | 91 | Ref. |
| M2/M3 | Negative | 2 | 5.9 (0.7, 49.6) 0.0996 |
| M1 | Positive | 29 | 2.7 (0.9, 8.5) 0.0836 |
| M2/M3 | Positive | 1 | 0.0 (0.0, Inf) 0.9982 |
| P interaction |  |  | 0.3714 |
| D33 MRD |  |  |  |
| <0.01% | Negative | 84 | Ref. |
| ≥0.01% | Negative | 9 | 1.7 (0.2, 14.6) 0.6058 |
| <0.01% | Positive | 14 | 0.8 (0.1, 7.0) 0.8714 |
| ≥0.01% | Positive | 5 | 2.4 (0.3, 20.2) 0.4130 |
| P interaction |  |  | 0.7765 |
| SCT |  |  |  |
| No | Negative | 90 | Ref. |
| Yes | Negative | 3 | 0.0 (0.0, Inf) 0.9987 |
| No | Positive | 28 | 2.4 (0.8, 7.2) 0.1183 |
| Yes | Positive | 3 | 0.0 (0.0, Inf) 0.9991 |
| P interaction |  |  | 0.9999 |
